# Effect of high‐fat diet on isometric, concentric and eccentric contractile performance of skeletal muscle isolated from female CD‐1 mice

**DOI:** 10.1113/EP091832

**Published:** 2024-05-09

**Authors:** Jason Tallis, Rob S. James, Emma L. J. Eyre, Sharn P. Shelley, Cameron Hill, Derek Renshaw, Josh Hurst

**Affiliations:** ^1^ Centre for Physical Activity, Sport & Exercise Science Coventry University Coventry UK; ^2^ Faculty of Life Sciences University of Bradford Bradford UK; ^3^ Randall Centre for Cell and Molecular Biophysics, New Hunt's House, Guy's Campus King's College London London UK; ^4^ Centre for Health & Life Sciences Coventry University Coventry UK

**Keywords:** isolated skeletal muscle, muscle lengthening, obesity, work‐loop

## Abstract

Despite evidence inferring muscle and contractile mode‐specific effects of high‐fat diet (HFD), no study has yet considered the impact of HFD directly on eccentric muscle function. The present work uniquely examined the effect of 20‐week HFD on the isometric, concentric and eccentric muscle function of isolated mouse soleus (SOL) and extensor digitorum longus (EDL) muscles. CD‐1 female mice were randomly split into a control (*n* = 16) or HFD (*n* = 17) group and for 20 weeks consumed standard lab chow or HFD. Following this period, SOL and EDL muscles were isolated and assessments of maximal isometric force and concentric work loop (WL) power were performed. Each muscle was then subjected to either multiple concentric or eccentric WL activations. Post‐fatigue recovery, as an indicator of incurred damage, was measured via assessment of concentric WL power. In the EDL, absolute concentric power and concentric power normalised to muscle mass were reduced in the HFD group (*P *< 0.038). HFD resulted in faster concentric fatigue and reduced eccentric activity‐induced muscle damage (*P *< 0.05). For the SOL, maximal isometric force was increased, and maximal eccentric power normalised to muscle mass and concentric fatigue were reduced in the HFD group (*P *< 0.05). HFD effects on eccentric muscle function are muscle‐specific and have little relationship with changes in isometric or concentric function. HFD has the potential to negatively affect the intrinsic concentric and eccentric power‐producing capacity of skeletal muscle, but a lack of a within‐muscle uniform response indicates disparate mechanisms of action which require further investigation.

## Abstract (highlights)

- **What is the central question of this study?**
  Is there a direct and muscle specific impact of dietary‐induced obesity on eccentric muscle function and to what extent is this comparable to effects on isometric and concentric activity?
- **What is the main finding and its importance?**
  Effects of dietary‐induced obesity on eccentric muscle function are muscle‐specific and have little relationship to changes in isometric or concentric function. Impaired eccentric function could present a significant risk to people living with obesity. Moreover, these data are valuable to inform the design of therapeutic strategies to prevent obesity‐impaired muscle health inferring distinct mechanisms account for contractile mode‐specific effects.

## 1 INTRODUCTION

Developing an understanding of the effect of obesity on skeletal muscle health is essential given that impaired function elevates the risk of developing metabolic complications and contributes to obesity‐associated comorbidities (Tallis et al., 2018, 2021). Moreover, poor skeletal muscle function is an independent risk factor for disease and all‐cause mortality (Ruiz et al., 2008). As such, there has been an increase in research examining the effects of obesity on skeletal muscle contractile function, which has been summarised in several reviews (Bollinger, 2017; Maffiuletti et al., 2007; Tomlinson et al., 2016). However, work to date has focused on developing an understanding of obesity effects on isometric and concentric contractile function, whilst consideration of effects on eccentric muscle actions is limited.

Despite some ambiguity, evidence from whole body in vivo studies demonstrates a trend for an obesity‐induced increase in the absolute force‐producing capacity of postural muscles (Lafortuna et al., 2005; Maffiuletti et al., 2007; Rolland et al., 2004), with little effect on non‐antigravity muscles (Hulens et al., 2001; Lafortuna et al., 2005; Rolland et al., 2004). This adaptation has been proposed to occur due to elevated demand placed on postural muscles by increased body weight (Maffiuletti et al., 2013; Tallis et al., 2018). However, when contractile function is expressed relative to body mass, muscle performance is substantially reduced in individuals with obesity (Hulens et al., 2001; Lafortuna et al., 2005; Maffiuletti et al., 2007), resulting in significant implications for physical function as each individual utilises their skeletal musculature to move and stabilise their body mass. Experiments examining the effect of high‐fat diet (HFD) on rodent isolated skeletal muscle function have been integral to developing the understanding of the impact of obesity on muscle function and have several distinct advantages (Tallis et al., 2018). Such approaches allow for more precise control of dietary composition, feeding duration and age, all of which are moderators to potential effects. Moreover, isolated skeletal muscle models allow a more precise assessment of muscle quality (muscle function relative to muscle size), examination of direct and muscle‐specific responses and assessment of muscle fatigue independent of the inertial mass of the articulating limb. Understanding the impact on muscle quality is particularly important in determining whether a reduction in the intrinsic force‐producing capacity of skeletal muscle contributes to obesity‐induced impaired physical function.

Research examining the effects of obesity on the contractile function of isolated skeletal muscle has been recently summarised (Tallis et al., 2018). A lack of methodological consistency in dietary composition (Ciapaite et al., 2015), intervention duration (Hurst et al., 2018), mode of contraction examined (Tallis et al., 2017) and differences in assessment temperatures (Tallis et al., 2022) prevent direct comparisons between studies. However, there is a clear body of evidence demonstrating that obesity causes a muscle‐specific reduction in muscle quality, which is likely related to muscle fibre type composition and in vivo biomechanical function (Ciapaite et al., 2015; Eshima et al., 2017; Shelley et al., 2024). To date, evidence examining the effect of HFD on isolated skeletal muscle function has focused on assessments of isometric and, to a lesser extent, concentric muscle function. No study has yet examined the effect of HFD on eccentric muscle function, an area identified as a priority for future work (Tallis et al., 2018). Eccentric muscle activity is essential for movement control, stabilisation, deceleration and shock absorbance (Choi, 2016; Dickinson et al., 2000). As such, effective eccentric muscle activity is pivotal in the completion of tasks of daily living (Chang et al., 2014; Davidson et al., 2013; LaStayo et al., 2003) where reduced eccentric function will exacerbate injury risk and impair physical function in individuals living with overweight and obesity.

Mechanistically, a HFD‐induced reduction in muscle quality has been attributed to impaired myogenesis (Akhmedov & Berdeaux, 2013; D'Souza et al., 2015), altered fibre type composition and metabolic profile (de Wilde et al., 2008; Denies et al., 2014; Shortreed et al., 2009; Yamauchi et al., 2002), degeneration in the process of excitation–contraction coupling, Ca^2+^ handling and altered cross‐bridge machinery (Bruton et al., 2002; Ciapaite et al., 2015; Funai et al., 2013). Interestingly, these mechanisms mirror those of muscle ageing (Demontis et al., 2013; Lightfoot et al., 2014; Miljkovic et al., 2015; Navarro et al., 2001), where increasing age results in a substantial decline in isometric and concentric muscle function, although eccentric function is relatively well preserved (Raj et al., 2010). Such discrepancies are likely explained by differences in skeletal muscle activation mechanics, where eccentric muscle actions are mechanically and physiologically distinct. Recent work has focused on understanding the action of stiffening of titin as an important protein involved with eccentric muscle force generation (Herzog, 2018; Hessel et al., 2017; Linke, 2023). This, in part, may suggest that HFD effects on eccentric muscle function may differ from those seen in other forms of muscle activity. Moreover, individuals living with obesity likely have a greater reliance on high‐force eccentric muscle actions given the need to control, stabilise and decelerate an elevated body mass. The increase in body mass may evoke favourable adaptations to preserve or enhance eccentric muscle function.

Experimental approaches using an isolated skeletal muscle model have distinct advantages allowing for accurate assessment of eccentric muscle function. Assessment of eccentric function in vivo is challenging and typically achieved with isokinetic dynamometry (Raj et al., 2010), and a lack of participant familiarity with high‐intensity eccentric muscle activity may lead to inconsistencies in results. Furthermore, given that high‐intensity eccentric muscle activity is associated with muscle damage (Call & Lowe, 2016; Proske & Morgan, 2001), there may be an intrinsic limitation in undertaking multiple attempts of maximally voluntary force. Such limitations are avoided when assessing eccentric muscle function using an isolated skeletal muscle. Given a limited understanding of the direct effect of HFD on eccentric muscle function and the importance of eccentric muscle action for physical function during daily living tasks, the present study harnessed the distinct advantages of an established isolated muscle model to uniquely examine the effect of 20 weeks HFD on the isometric, concentric and eccentric muscle function of isolated mouse soleus (SOL) and extensor digitorum longus (EDL) muscles.

## 2 METHODS

### 2.1 Ethical approval

The experimental procedures and the use of animals in this study were approved by the Coventry University Ethics Committee (P60244). Animals used in this study were kept in accordance with the principles of laboratory animal care (NIH publication No. 86‐23, revised 1985).

### 2.2 Animal morphology and muscle preparation

Four‐week‐old CD‐1 female mice (*n* = 33, Charles River, Harlow, UK) were randomised into either a HFD (*n* = 17) or a control group (*n* = 16). Our research group have contributed to an improved understanding of contractile mechanics in healthy weight and HFD‐fed female CD‐1 mice and as such, this strain and sex were selected to allow comparison to previous work. For the next 20 weeks, all mice were kept in groups of 8–10 in 12:12‐h light–dark cycles with ad libitum access to water and standard lab chow (SDS maintenance diet, Dietex International, Essex, UK; calories provided by protein 17.5%, fat 7.4%, carbohydrate, 75.1%; gross energy 3.52 kcal/g; metabolisable energy 2.57 kcal/g). In addition to the standard lab chow, mice in the HFD group had ad libitum access to a high‐fat laboratory forage diet (PicoLab Natural Sunflower; calories provided by protein 18.0%, fat 63.7%, carbohydrate, 18.4%; gross energy 5.2 kcal/g; metabolisable energy 3.8 kcal/g). This dietary protocol has been used to induce obesity in previous work (Hurst et al., 2018; Tallis et al., 2017, 2022).

At 24 weeks, animals were sacrificed by cervical dislocation (in accordance with British Home Office Animals [Scientific Procedures] Act 1986, schedule 1). Mice were then weighed to the nearest 0.1 g using an electronic balance (Fisher Scientific 15385113, Fisher Scientific, Loughborough, UK) and snout‐to‐anus length was measured using electronic callipers (Fisher Scientific 3417). The gonadal fat pad was dissected and weighed to give fat pad mass (FPM) as an indicative measure of whole‐body adiposity (Rogers & Webb, 1980) for each individual.

Either whole SOL or EDL muscle was rapidly dissected from both the right and left hind limbs, respectively, in refrigerated (1−3°C) oxygenated (95% O_2_–5% CO_2_) Krebs–Henseleit solution (mM: NaCl 118; KCl 4.75; MgSO_4_ 1.18; NaHCO_3_ 24.8; KH_2_PO_4_ 1.18; glucose 10; CaCl_2_ 2.54 in each case; pH 7.55 at room temperature before oxygenation). For both the SOL and EDL, aluminium foil T‐clips were wrapped around the distal tendon, as close to the muscle as possible, with a fragment of bone left attached to the proximal tendon. The dissection of one SOL and one EDL muscle from each animal resulted in the formation of the following four experimental groups: Control Concentric; Control Eccentric, HFD Concentric; HFD Eccentric (*n* = 8 or greater in each case).

### 2.3 Assessment of contractile performance

Assessment of contractile performance followed protocols previously published by our research group (Hill et al., 2018; Tallis et al., 2017). In brief, contractile performance was measured using custom‐designed equipment. Each muscle was placed in a Perspex chamber filled with oxygenated Krebs–Henseleit solution maintained at 37 ± 0.2°C. Krebs–Henseleit solution was circulated through the Perspex chamber, using peristaltic pumps (120, Watson & Marlow, Falmouth, UK), from a central reservoir kept in a heater/cooler (Grant LTD6G, Grant Instruments Ltd, Shepreth, UK) to maintain the target temperature. Temperature within the chamber was continuously monitored using a digital thermometer (Checktemp C, Harvard Apparatus, Cambridge, UK). Using the intact bone left at the proximal end, and the aluminium foil T‐Clip attached at the distal end, each muscle was placed in the Perspex chamber and attached, via crocodile clips, to a force transducer (UF1, Pioden Controls Ltd, Ashford, UK) at one end, and a motor arm (V201, Ling Dynamic Systems, Royston, UK) at the other. Position of the motor arm was detected by a linear variable displacement transducer (DFG5.0, Solartron Metrology, Bognor Regis, UK). The muscle was electrically stimulated to produce force via platinum electrodes submerged in the Krebs–Henseleit solution and placed parallel to the muscle. Stimulation and length change parameters were controlled using custom‐written software (CEC Testpoint, Measurement Computing, Norton, MA, USA) via a D/A board (KPCI3108, Keithley Instruments, Cleveland, OH, USA) on a standard desktop personal computer.

### 2.4 Isometric assessments

Following a 10‐min stabilisation period, muscle length and stimulation parameters (typically 12−16 V for SOL and 14−18 V EDL; fixed stimulation amplitude of 160 mA and pulse width of 1.2 ms) were adjusted to produce a maximal isometric twitch. Electrical stimulation was controlled and delivered by an external power source (PL320, Thurlby Thandar Instruments, Huntingdon, UK), and each twitch response was measured using a storage oscilloscope (2211, Tektronix, Marlow, UK). Using the optimal length and stimulation parameters from the isometric twitch assessment, and a fixed duration of electrical stimulation (250 ms for EDL, 350 ms for soleus), stimulation frequency was adjusted (typically 120–140 Hz for soleus and 200–220 Hz for EDL) to evoke a maximal tetanus response. Each tetanus activation was separated by a 5‐min rest period. The optimal muscle length (*L*_0_) used in the isometric tetanus assessments was measured using an eyepiece graticule fitted to a microscope. Estimates of mean fibre length were determined as 85% of the physical length for SOL and 75% for EDL (James et al., 1996).

### 2.5 Concentric work loop assessments

Concentric muscle power output was then measured using the work loop (WL) technique. Previous work has indicated that this method provides a closer representation of the contractile mechanics used by in vivo power‐producing muscles (James et al., 1995, 1996; Josephson, 1993). Here, each muscle was subjected to a symmetrical sinusoidal length change around the previously determined *L*_0_. The onset and duration of the electrical stimulation were manipulated to evoke force production during muscle shortening. Instantaneous force and velocity were sampled, throughout the length change cycle, at a rate of 10 kHz, and plotted against each other to form a WL. Concentric net work was calculated as the positive work produced during shortening, minus the work required to re‐lengthen the muscle. The electrical stimulation was delivered to the muscle at the pre‐determined optimal cycle frequency strain amplitude and stimulus burst duration. The amplitude of the length change waveform (i.e., the strain), the stimulus phase (timing of stimulation relative to maximal length during the WL) and stimulus burst duration were optimised to elicit maximal net work at fixed cycle frequencies of 5 and 10 Hz for SOL and EDL, respectively. These cycle frequencies are indicative of maximal WL power for these muscles (James et al., 1995). Typically, a strain of 0.10 of *L*_0_ was used to elicit maximal net work for each muscle, indicating that the muscle initially lengthened by 5%, shortened by 10% and re‐lengthened by 5% back to *L*_0_. Typical phase shifts of −10 and −2 ms for SOL and EDL, respectively, and stimulus burst durations of 50 and 65 ms for SOL and EDL, respectively, were used to produce maximal net work. The stimulation timing was manipulated to evoke maximal net work. Put simply, under‐stimulation would result in a reduction in positive work, and over‐stimulation causes an increase in negative work as the muscle fails to relax sufficiently before being re‐lengthened. Sub‐optimal stimulation or length change parameters have a profound effect on WL power (cycle frequency × net work). During each assessment, each muscle was stimulated to produce four WLs. Each WL assessment was separated by a 5‐min rest period.

### 2.6 Sustained concentric and eccentric WL protocol

**FIGURE 1:**
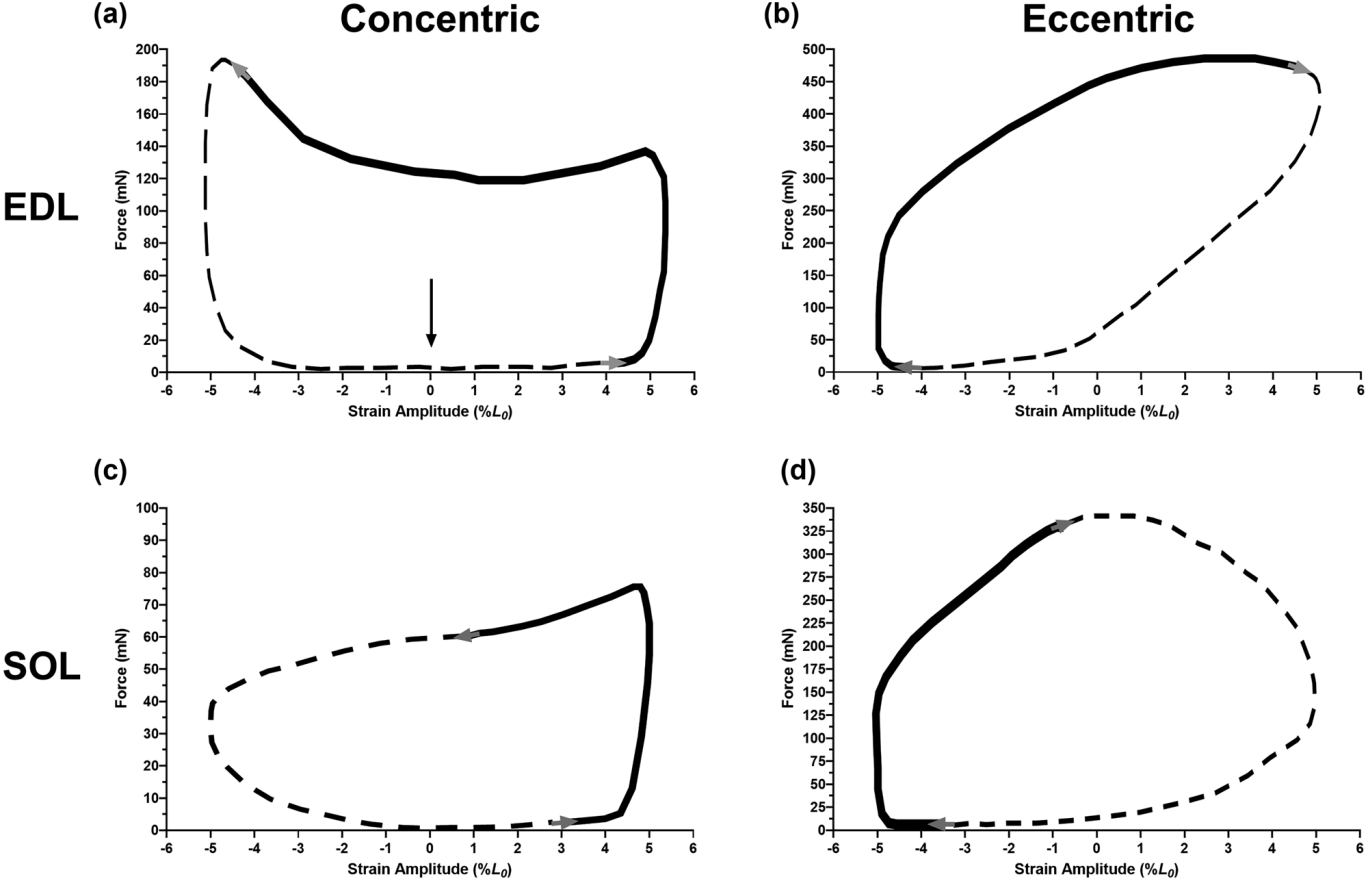
Typical concentric (a, c) and eccentric (b, d) work loop shapes for isolated mouse EDL (a, b) and SOL (c, d) muscle optimised for maximal work at 10 and 5 Hz cycle frequency, 240 and 140 Hz stimulation frequency and 5% strain amplitude, respectively. Initiation of all work loops starts at *L*_0_ as indicated by the arrow in (a). Concentric work loops proceed in an anti‐clockwise direction and eccentric work loops in a clockwise direction, as indicated by grey arrows in each panel. The bold continuous line in each loop represents the stimulation duration (50 and 65 ms for the EDL and SOL, respectively, for concentric work loops; 55 and 72 ms for the EDL and SOL, respectively, for eccentric work loops).

The isometric and concentric WL power assessments outlined above were completed for all muscles. Following a 10‐min rest period, each muscle was subjected to either a concentric or an eccentric fatiguing protocol. Mice in the concentric experimental groups were subjected to a fatiguing protocol consisting of 50 consecutive concentric WLs at the previously determined optimal parameters. Mice in the eccentric experimental groups underwent an eccentric fatiguing protocol in the same manner as that described in our previous work (Hill et al., 2018). Assessment of acute maximal eccentric WL power, in the same manner as previously described for the assessment of maximal concentric WL power, was not conducted due to the potential for eccentric activity to cause irreparable damage. Therefore, contractility parameters were fixed for each muscle for the sustained eccentric WL protocol. Given that 5‐ and 10‐Hz cycle frequencies for SOL and EDL, respectively, typically represent the contraction velocities needed to elicit maximal concentric WL power, these cycle frequencies and previously determined optimal strain were used in the assessment of eccentric WL power. Muscles were subjected to 50 consecutive eccentric WLs. An eccentric WL was elicited by using a strain of −0.10 of *L*_0_, so that each muscle passively shortened, followed by stimulation through lengthening, followed by passive re‐shortening back to *L*_0_. A phase shift of −10 and −2 ms for SOL and EDL was maintained to ensure stimulation was provided prior to the muscle reaching its shortest length. A fixed burst duration of 72 and 55 ms was used for SOL and EDL, respectively, to ensure that the muscle was sufficiently stimulated throughout the lengthening phase. The second WL of the eccentric fatigue protocol was used to calculate the maximum eccentric WL power. Typical WL shapes for optimised maximal concentric and eccentric power output are provided in Figure 1. For each of the fatigue protocols, WL power was plotted as a percentage of the maximal value obtained during the stimulation protocol.

### 2.7 Recovery protocol

The ability of each muscle to recover from the respective fatigue protocol was measured by stimulating each muscle to produce four concentric WLs, in the same manner as previously described, at 10‐min intervals for a total duration of 30 min after the fatigue protocol had been completed. In each case, data were plotted as a percentage relative to pre‐fatigue concentric WL power output.

### 2.8 Muscle mass dimensions and calculations

The muscle was detached from the equipment, and tendons and bone removed. Each muscle was then blotted on absorbent paper, to remove excess Krebs–Henseleit solution, and placed on an electronic balance (Mettler Toledo B204‐S, Zurich, Switzerland) to determine wet muscle mass. Mean muscle cross‐sectional area was calculated from *L*_0_, muscle mass and an assumed muscle density of 1060 kg/m^3^ (Mendez & Keys, 1960). Isometric stress (kN m^2^) was calculated as maximal tetanic force divided by mean muscle cross‐sectional area. Absolute power output (watts) was calculated as the product of net work and cycle frequency. Power output normalised to muscle mass (W kg^−1^ muscle mass) was calculated as absolute power output divided by muscle mass.

### 2.9 Statistical analysis

Following appropriate checks of normality and homogeneity, whole animal morphology, skeletal muscle morphology, isometric function and maximal WL PO data were analysed using Student's *t*‐test for independent samples. On the small number of occasions where tests for normality and homogeneity were violated, data were analysed using a Mann–Whitney test. Muscle performance over the sustained WL protocol was plotted as a percentage of maximal WL power and cumulative work was also determined. This approach allows both between‐group differences in the rate of fatigue to be compared and the amount of work performed over the duration of the stimulation protocol to be determined. As per our previous work (Shelley et al., 2022, 2024), the effect of HFD on the rate of fatigue and cumulative work was assessed via statistical parametric mapping (SPM) using the SPM−1D package (Todd Pataky, v.M0.1) via MATLAB (The MathWorks Inc, R2018b, Natick, MA, USA) (Pataky, 2010). A two‐sample SPM[t] (two‐sided *t*‐test) was conducted to assess the effect of treatment. SPM calculates the *t‐*statistic for every data point, but instead of calculating a *P*‐value for every data point, inferential statistics are based on random field theory and thus maintain a constant error of α (Pataky et al., 2013). Where clusters crossed the critical threshold, this indicated a significant difference at *P* ≤ 0.05. Recovery was assessed using a mixed model ANOVA, with time as the within‐group factor and treatment (HFD vs. control) as the between‐group factor. Significant main effects were explored using Bonferroni‐corrected pairwise comparisons. Partial eta squared (η_p_^2^) was calculated as an estimate of effect size and was interpreted as small (>0.01), medium (>0.06) or large (>0.14) (Richardson, 2011). Where relevant, Cohen's *d* was calculated and corrected for bias using Hedge's *g* (Lakens, 2013). Hedge's *g* effect size was interpreted as trivial (<0.2), small (<0.6), moderate (<1.2) or large (>1.2) (Hopkins et al., 2009). Other than SPM, statistical analysis was performed using SPSS Statistics 26.0 (IBM Corp., Armonk, NY, USA) and graphical presentation of data was performed using GraphPad Prism (Version 8.3.1, GraphPad Software, San Diego, CA, USA). Statistical significance was a priori set at an α‐level of 0.05. Data are presented as means ± SD.

## 3 RESULTS

### 3.1 Whole animal and skeletal muscle morphology

Body mass, snout‐to‐anus length, gonadal FPM and gonadal FPM as a percentage of body mass were greater in the HFD group compared to the control group (Table 1; *P *< 0.001; *g *> 1.12).

**TABLE 1:** The effect of 20 weeks HFD on whole animal morphology.

	Body mass (g)	Body length (mm)	FPM (g)	FPM/body mass (%)
Control (*n* = 16)	34.6 ± 6.4	101.7 ± 6.0	1.5 ± 1.2	4.1 ± 2.8
HFD (*n* = 17)	46.9 ± 9.2*	107.2 ± 3.6*	5.4 ± 2.8*	11.0 ± 4.2*
*P*	<0.001	<0.001	<0.001	<0.001
*g*	1.56	1.12	1.79	1.94

Data represented as means ± SD; *g* is Hedge's *g* corrected effect size. *Significant differences between Control and HFD treatments (*P *< 0.05). FPM, gonadal fat pad mass.

Muscle mass of the HFD SOL was greater than that of the control (Table 2; *P *< 0.001; *g* = 1.70). Muscle mass of the EDL and muscle length for both SOL and EDL were unaffected by the HFD treatment (Table 2; *P *> 0.117; *g* < 0.57).

**TABLE 2:** The effect of 20 weeks HFD on skeletal muscle morphology.

	Muscle mass (mg)	Muscle length (mm)
SOL		
Control (*n* = 16)	8.9 ± 1.2	9.9 ± 0.5
HFD (*n* = 16)	11.4 ± 1.7*	9.9 ± 0.6
* P*	<0.001	0.897
* g*	1.70	0.02
EDL		
Control (*n* = 16)	12.3 ± 1.0	9.0 ± 0.5
HFD (*n* = 17)	12.5 ± 1.4	9.3 ± 0.6
* P*	0.642	0.118
* g*	0.18	0.56

Data represented as mean ± SD; *g* is Hedge's *g* corrected effect size. *Significant differences between Control and HFD treatments (*P *< 0.05).

### 3.2 Maximal isometric force and power output

**FIGURE 2:**
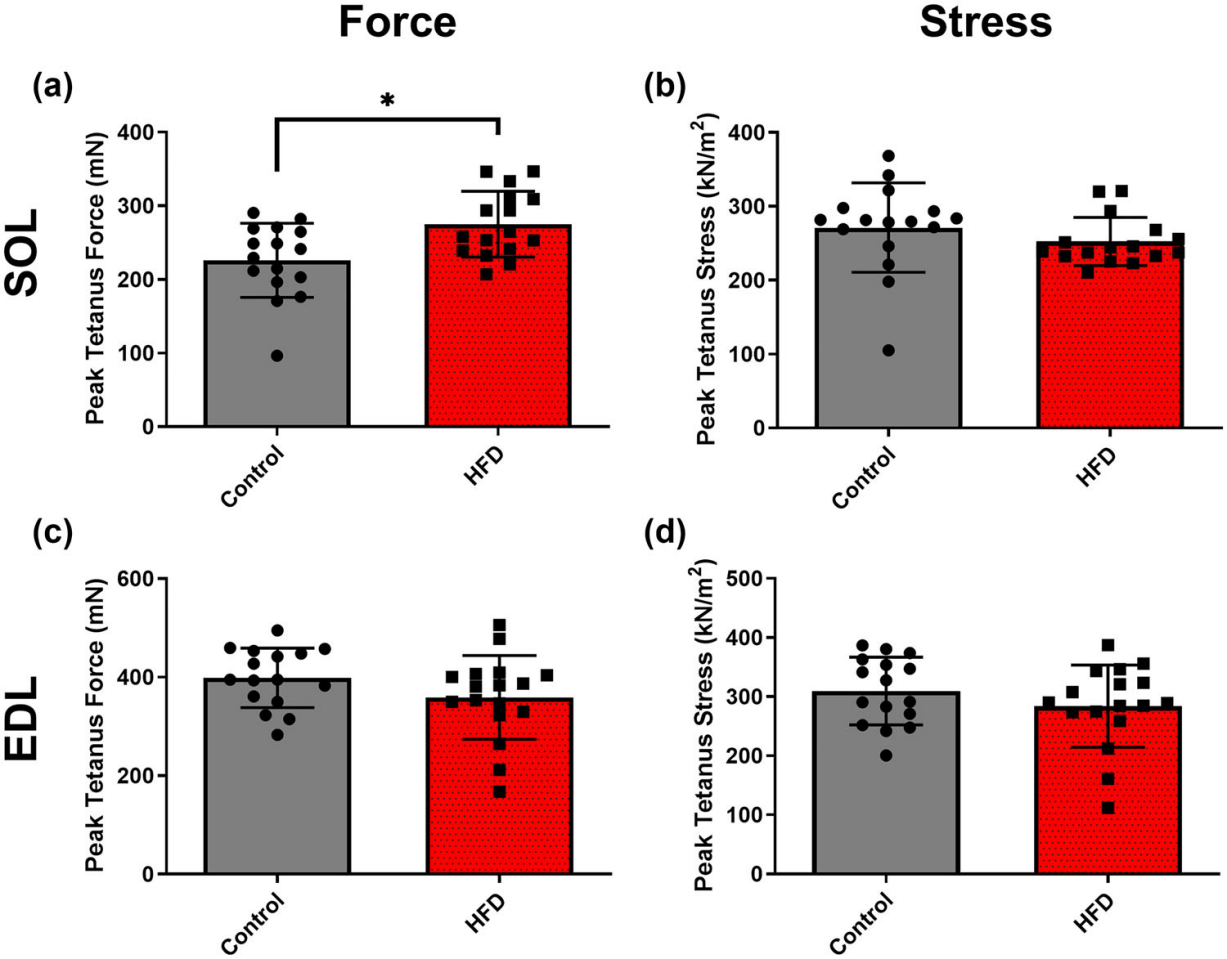
The effect of 20 weeks of a HFD on maximal isometric force and stress of mouse soleus (a, b) and EDL muscle (c, d). Data presented as means ± SD; *n* = 16–17 in each group. *Significant difference between groups.

Maximal isometric force of the SOL was greater in the HFD group compared with the control (Figure 2a; *P* = 0.007; *g *= 1.03). Maximal isometric force of the EDL (Figure 2c; *P* = 0.134; *g *= 0.54) and maximal isometric stress of the both the SOL (Figure 2b; *P* = 0.066; *g *= 0.39) and the EDL (Figure 2d; *P* = 0.257; *g *= 0.46) were unaffected by HFD.

**FIGURE 3:**
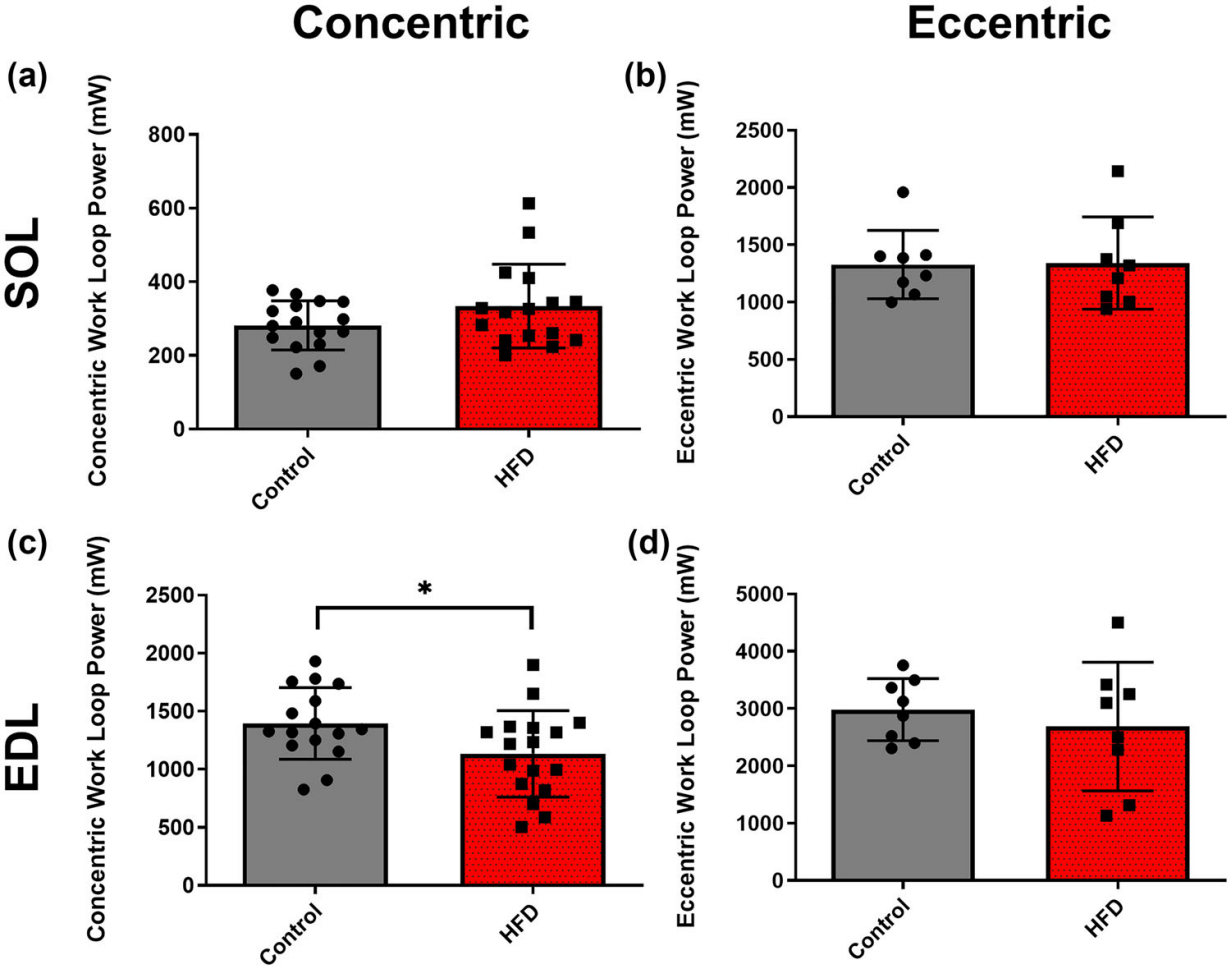
The effect of 20 weeks of a HFD on maximal absolute concentric and eccentric work loop power output of mouse soleus (a, b) and EDL muscle (c, d). Data presented as means ± SD; *n* = 16–17 for concentric, *n* = 8 for eccentric. *Significant difference between groups.

Maximal absolute concentric WL power of the EDL was lower in the HFD group compared to control (Figure 3c; *P* = 0.037; *g *= 0.759). Maximal absolute concentric power of the SOL (Figure 3a; *P* = 0.386; *g *= 0.556) and maximal absolute eccentric power of both the SOL (Figure 3b; *P* = 0.942; *g *= 0.039) and the EDL (Figure 3d; *P* = 0.516; *g *= 0.333) were unaffected by HFD.

**FIGURE 4:**
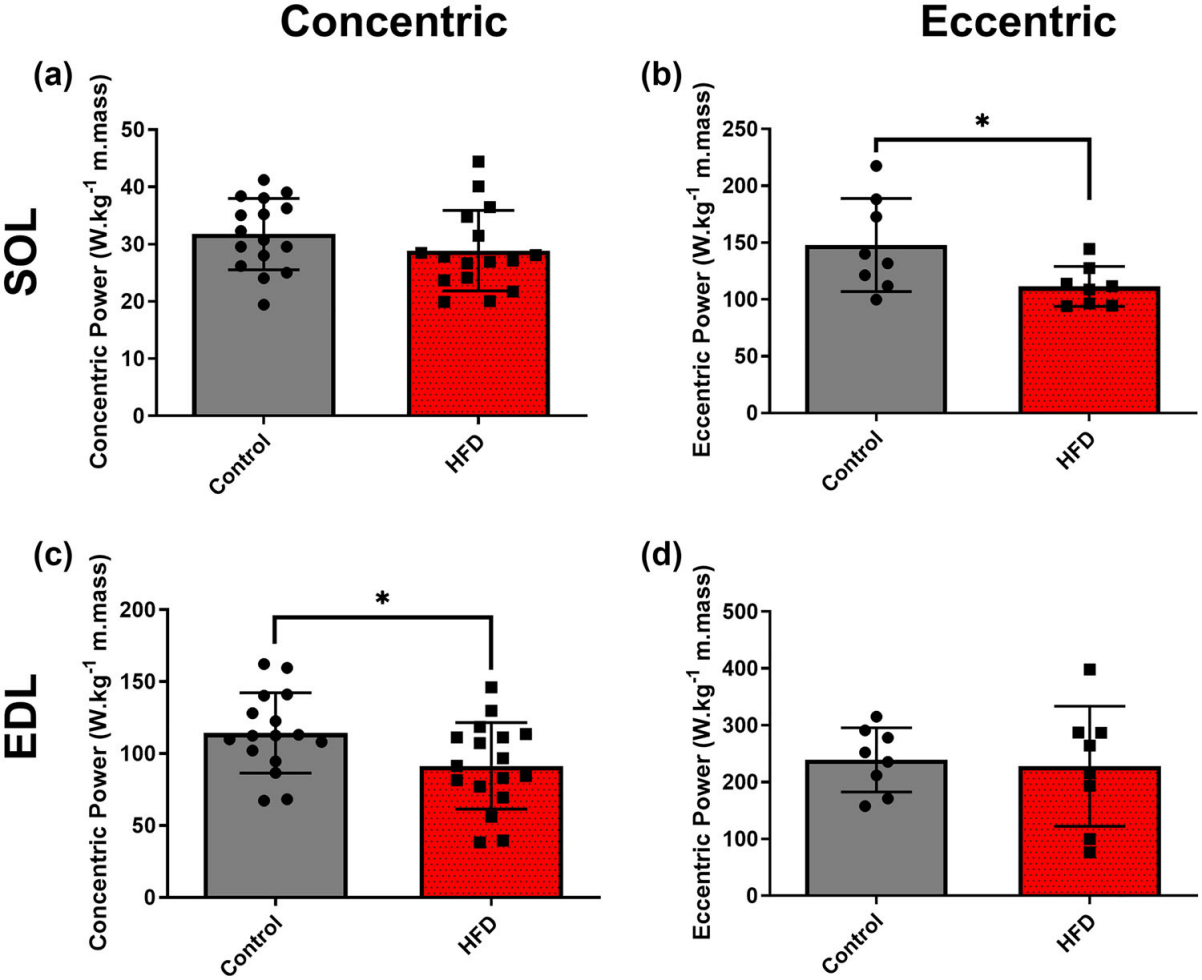
The effect of 20 weeks of a HFD on normalised maximal work loop power output of mouse soleus (a, b) and EDL muscle (c, d). Data presented as means ± SD; *n* = 16–17 for concentric, *n* = 8 for eccentric. *Significant difference between groups.

When normalised to muscle mass, maximal eccentric WL power of the SOL (Figure 4b; *P* = 0.036; *g *= 1.157) and concentric WL power of the EDL (Figure 4c; *P* = 0.032; *g *= 0.785) were reduced in the HFD group compared to control. Normalised concentric power of the SOL (Figure 4b; *P* = 0.229; *g *= 0.435) and eccentric power of the EDL (Figure 4d; *P* = 0.792; *g *= 0.134) were unaffected by the HFD treatment.

### 3.3 Sustained concentric and eccentric work loop power and recovery

**FIGURE 5:**
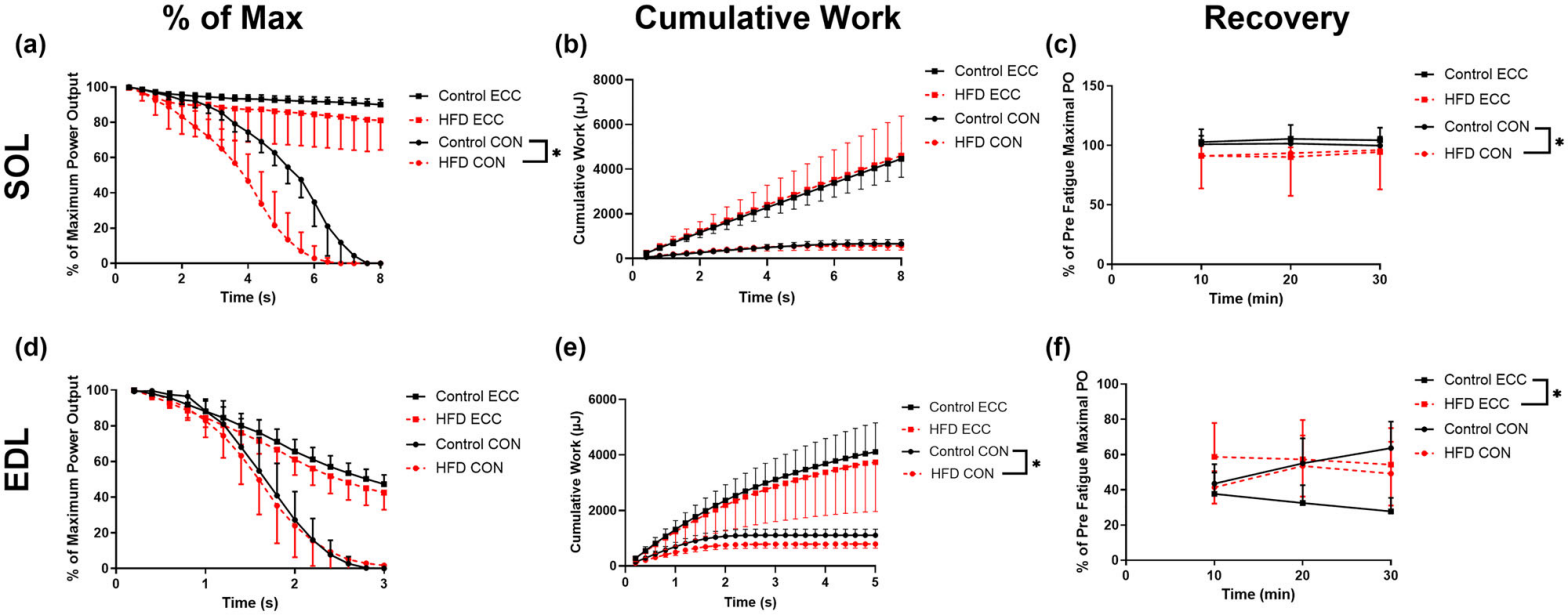
The effect of 20 weeks of a HFD on concentric (Con) and eccentric (Ecc) rate of fatigue, cumulative work and concentric recovery of maximally stimulate mouse SOL (a–c) and EDL muscle (d–f). Data presented as means ± SD; *n* = 8–9 in each group. *Significant difference between groups.

An SPM *t*‐test indicated a significant main effect of treatment for sustained concentric WLs of the SOL, where the percentage decline in power output was greater in the HFD group between 4 and 6 s compared to control (Figure 5a; *P *< 0.05). For concentric cumulative work of the EDL, there was also a main effect of treatment (Figure 5d; *P *< 0.05), with control producing greater cumulative work than control from 0.8 s. There were no other effects of treatment (Figure 5; *P *> 0.5).

Recovery of the SOL following sustained eccentric WLs did not differ between treatments (Figure 5c; *P* = 0.297; η_p_^2^ = 0.083). However, recovery of the SOL following sustained concentric WLs was greater in the control group compared to HFD (Figure 5c; *P* = 0.022; η_p_^2^ = 0.321). In both cases, there was no main effect of time (Figure 5c; *P *> 0.070; η_p_^2^ < 0.185) and no treatment × time interaction (Figure 5c; *P *> 0.097; η_p_^2^ < 0.165).

**FIGURE 6:**
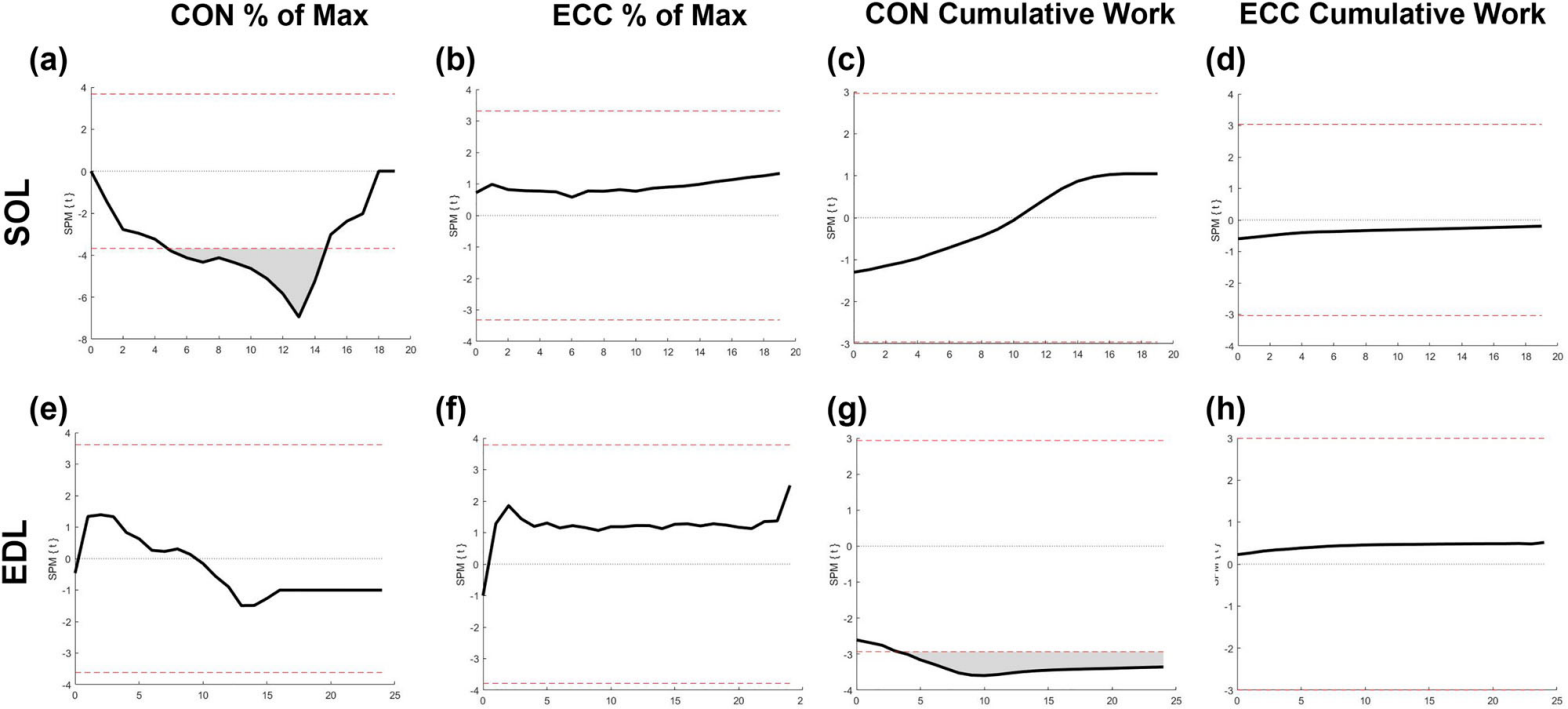
Statistical parametric mapping *t*‐test result for concentric (Con) and eccentric (Ecc) rate of fatigue and cumulative work for SOL (a–d) and EDL (e–h) muscle.

Recovery of the EDL following sustained eccentric WLs was greater in the HFD group compared to control (Figure 5e; *P* = 0.009; η_p_^2^ = 0.416). There was also a main effect of time (Figure 5e; *P* = 0.007; η_p_^2^*^^* = 0.315), where WL power was reduced at 30 min compared to 10 min (Figure 5e; *P* = 0.046; *g *= 0.170). Recovery of the EDL following sustained concentric WLs was not affected by treatment (Figure 5e; *P* = 0.327; η_p_^2^ = 0.069). There was a significant main effect of time (Figure 5e; *P* = 0.001; η_p_^2^*^^* = 0.420), where power after 20 and 30 min was higher than that at 10 min following the sustained WL protocol (Figure 5e; *P *< 0.004; *g *< 0.420). In both cases, there was no treatment × group interaction (Figure 5e; *P *> 0.128; η_p_^2^*^^* < 0.137). The figures generated as part of SPM are presented as supplementary information (Figure 6).

## 4 DISCUSSION

The present work examined the direct effect of HFD on isometric, concentric and eccentric muscle function, and as such, presents novel data with respect to the effect of HFD on eccentric muscle function. The data indicate, for the first time, that HFD effects on eccentric muscle function are muscle‐specific and have little relationship to HFD‐associated changes in either isometric or concentric muscle function. Despite a HFD‐induced reduction in the concentric function of the EDL, eccentric function is well maintained. Conversely for the SOL, eccentric power output normalised to muscle mass was reduced following HFD treatment and maximal concentric power output was maintained. Although maximal concentric power output was unaffected, HFD resulted in faster fatigue of the SOL. Interestingly, recovery of EDL following sustained eccentric WL activations was increased in the HFD, which may indicate a HFD‐induced reduction in muscle damage evoked by high‐force eccentric muscle actions.

### 4.1 Maximal force and power

With respect to HFD effects on concentric muscle function, findings in the present work align with the trends seen in previous work on mouse skeletal muscle (Hurst et al., 2018; Shelley et al., 2024; Tallis et al., 2017, 2022). The HFD‐induced increase in an absolute isometric force of the SOL has previously been reported as a positive adaptation of this postural muscle to elevated body mass. However, the magnitude of this increase (16.6%) does not align with the magnitude of increased body mass (30.2%), so such an effect is unlikely to preserve in vivo locomotor function. The lack of a similar change in the absolute WL power of SOL aligns with previous work (Hurst et al., 2018; Tallis et al., 2017). Such findings initially indicate that HFD effects vary across contractile modalities. Although isometric function of the EDL appeared to be unaffected by HFD, absolute WL power and power normalised to muscle mass were reduced. It has been proposed that HFD effects on fast‐twitch muscle may be more severe given the reduced capacity to metabolise lipid compared to muscle with a more predominant slow‐twitch phenotype, resulting in greater lipid accumulation (Hurst et al., 2018).

The current study is the first to examine the effect of HFD on the eccentric function of isolated muscle. Interestingly, there appears to be little relationship between HFD‐associated changes in isometric, concentric and eccentric muscle function in either muscle, with HFD effects on contractile modality further differing between specific muscles. Despite a substantial decline in the concentric WL function of the EDL, absolute eccentric WL power and WL power as a function of muscle mass were similar between the HFD and control groups. In contrast, whilst the isometric and concentric function of the SOL was preserved with HFD, eccentric function was significantly reduced, with the present study providing data for the first time that indicate HFD may cause a direct reduction in eccentric muscle quality. Although the mechanisms remain unexplored, such results indicate a discrepancy between the principal processes resulting in HFD‐associated changes in concentric function compared to those resulting in changes in eccentric muscle function.

Typically a change in skeletal muscle fibre type composition, altered protein synthesis, changes in processes associated with excitation–contraction coupling and metabolic processes involved with ATP generation have been cited as primary mechanisms causing a reduction in isometric and concentric contractile performance following HFD consumption in rodents (Akhmedov & Berdeaux, 2013; Ciapaite et al., 2015; de Wilde et al., 2008; Denies et al., 2014). Despite this, there is a lack of evidence directly linking such proposed mechanisms to HFD‐related changes in contractile function. However, maybe somewhat unsurprisingly, mechanistic changes have been demonstrated to be muscle‐specific (Ciapaite et al., 2015; Messa et al., 2020; Shortreed et al., 2009; Thomas et al., 2014). Whilst these mechanisms may adequately explain HFD‐induced changes in concentric function, data in the present study indicate that effects on eccentric function are not concurrent, inferring HFD‐induced changes in physiological processes beyond those currently recognised in the literature.

Whilst concentric power was reduced, eccentric power of the EDL was not affected by HFD, indicating that processes responsible for producing resistance to muscle lengthening are either preserved or augmented to offset a loss in resistance to lengthening as a result of impaired cross‐bridge function. Support for this can be drawn from recent work by Delgado‐Bravo et al. (2023), demonstrating a HFD‐induced increase in passive force of rat gastrocnemius at long muscle lengths when subjected to a maximal isometric force–length relationship assessment. Physiological processes involved in eccentric muscle actions are less well understood; however, the preserved eccentric power of the EDL seen in the present study may be explained by increased stiffness induced by elevated muscle lipid deposits, increased collagen content (Delgado‐Bravo et al., 2023) and/or a maintenance or improvement in the action of Ca^2+^‐activated titin. There is growing evidence indicating Ca^2+^ released during muscle activation binds directly to titin resulting in stiffening, thus increasing resistance to stretch (Herzog, 2018; Hessel et al., 2017).

Conversely, eccentric function of the SOL was impaired by HFD despite previous evidence indicating increased lipid accumulation (Baek et al., 2018; Eum et al., 2020) and either preserved or increased collagen (Delgado‐Bravo et al., 2023; Pincu et al., 2015). Given that concentric function was preserved, reduced eccentric function may be explained by a HFD‐induced impairment of titin function. The discrepancy between EDL and SOL may be explained by a titin isoform‐specific effect. Whilst plausible based on the available data, this speculative account should be the focus of future work, where particular focus on HFD effects on the Cronos titin isoform may be a sensible starting point for investigation given near exclusive expression in fast‐twitch muscle (Linke, 2023).

### 4.2 Fatigue resistance and recovery

The effect of HFD on the rate of concentric fatigue of the SOL and EDL aligns with our previous work examining a similar HFD treatment duration (Tallis et al., 2017). Despite the distinct benefits of isolated skeletal muscle models for evaluating the direct effects on fatigue resistance, studies exploring this concept are sparse, particularly with respect to measures of muscular power. This study provides novel insight into HFD effects on fatigue by examining performance during sustained eccentric power‐producing activations and by examining HFD effects on concentric and eccentric cumulative work.

HFD reduced concentric fatigue resistance of the SOL, but had little effect on the EDL in agreement with previous work on mice (Tallis et al., 2017). Results of the present study further align with recent evidence showing a HFD‐induced reduction in the cumulative work of the EDL. However, a lack of effect on the cumulative work of the SOL differs from results presented by Shelley et al. (2024). This may in part be attributable to differences in feeding regimes and durations which have been shown to influence the detection of a HFD effect in mice (Ciapaite et al., 2015; Hurst et al., 2018). However, when findings of the present work are considered collectively with previous observations, there is now a strong basis of evidence demonstrating that HFD causes a faster loss of concentric power and/or that the amount of work performed over a repeated bout of actions is reduced. Consequently, these data indicate that reduced exercise capacity seen in obese humans in vivo (Pataky et al., 2014) cannot be solely attributed to elevated body mass, but is also partly explained by reduced fatigue resistance directly at the muscle.

During sustained eccentric WL activations, neither the rate of fatigue nor cumulative work of the SOL or EDL was affected by treatment, further compounding the contractile mode‐specific effects of HFD treatment. Despite a lack of direct muscle effects on these measures, the in vivo implications should not be understated. Body mass of the HFD group was substantially higher than that of the control group, so if working in vivo to fulfil locomotor tasks, muscles of the HFD group would have to work at a relatively higher intensity than controls to maintain a fixed workload due to the elevated body inertia. The elevated body mass is likely to further exacerbate the in vivo consequences of impaired concentric fatigue resistance as explained in previous work (Tallis et al., 2018).

It has been proposed that the elevated body mass would induce greater eccentric muscle function given a higher in vivo demand for high‐force eccentric muscle activation to control, stabilise and decelerate an elevated body mass (Tallis et al., 2018). Whilst this might be the case for EDL, given that absolute eccentric power output was maintained and concentric power decreased, the lack of any further HFD‐induced changes in cumulative work of the EDL and absolute power output and cumulative work of the SOL, these data infer a lack of support for this hypothesis. This may, therefore, infer that either the frequency and intensity of eccentric muscle activity does not differ between obese and healthy‐weight groups or there is a lack of ability to adapt, which may be underpinned by HFD‐impaired myogenesis.

One particular risk associated with high‐intensity eccentric muscle activity is damage to the contractile apparatus and consequently a reduction in exercise capacity (Lima & Denadai, 2015; Proske & Morgan, 2001). Such effects would present greater risks to individuals living with obesity, given an already reduced functional capacity, and are of particular importance given the recent focus on the benefits of eccentric training for individuals with chronic diseases, in particular, obesity (Hoppeler, 2016; Julian et al., 2018; Nikolaidis et al., 2008; Paschalis et al., 2011). Concentric WL power for 30 min following the sustained WL protocol was monitored in each muscle to evaluate treatment effects on muscle damage. Recovery of the SOL following sustained eccentric WLs was not affected by treatment; however, HFD caused reduced recovery following sustained concentric WLs. It is proposed that this is metabolic fatigue rather than damage, given that at the final sampling point, SOL had recovered to 99.8% and 96.1% in the control and HFD groups, respectively. This difference is likely attributable to the faster fatigue seen in the HFD group.

Interestingly, recovery of the EDL following sustained concentric WLs was unaffected, whilst recovery following sustained eccentric WLs was improved in the HFD group. The negative trajectory of recovery seen in the EDL subject to sustained eccentric WLs is indicative of muscle damage and differs from the increased performance over time following recovery from concentric WLs, which is similar to previous work (Hill et al., 2018). Improved recovery may indicate a protective effect of HFD on eccentric activity‐induced muscle damage in the EDL. Hessel et al. (2017) stated that it is high‐intensity unaccustomed muscle activity that results in muscle damage. As such, it could be argued that a further reliance on high‐force eccentric muscle action in obese individuals may account for a reduction in eccentric activity‐induced muscle damage. Whether this is the case in caged rodents is difficult to determine. Given the limited understanding of eccentric muscle physiology and more specifically HFD effects on these processes, it is difficult to fully rationalise these results, indicating a need for future research.

A protective effect from damage induced by high‐intensity eccentric muscle activity is a timely addition to the literature, given the recent evidence indicating that sustained moderate‐intensity eccentric muscle activity may be more beneficial for decreasing fat mass compared to concentric activity at the same intensity (Julian et al., 2018), which has been attributed to an elevated post‐exercise respiratory exchange ratio (Hoppeler, 2016; Paschalis et al., 2013). In addition, eccentric exercise has been demonstrated to evoke greater increases in lean mass compared to concentric activity (Julian et al., 2018; LaStayo et al., 2009), although it is established that this needs further investigation, particularly in individuals living with obesity.

### 4.3 Limitations and future direction

The sinusoidal length change waveforms used in the WL protocol provide an approximation of in vivo cyclical activities, but are a simplification of the muscle‐specific length change waveforms used in vivo (Dickinson et al., 2000). Such unperturbed length change waveforms are approximate muscle actions during activities such as steady walking and running. Future work should be undertaken to understand if these findings translate to perturbated muscle length cycles that reflect unsteady locomotory performance (Sponberg et al., 2023). Furthermore, fibre stimulation and length change waveform may be manipulated during fatiguing contractions in vivo in order to optimise net work (Wakeling & Rozitis, 2005). Fatigue is associated with dysfunctional sarco(endo)plasmic reticulum Ca^2+^‐ATPase (SERCA) and as a result muscle relaxation time is prolonged (Allen et al., 2008; Nogueira et al., 2013). Using a concentric WL as an example, if the relaxation rate of the muscle decreases so that it is active as it begins to lengthen (i.e., is producing eccentric work), the duration of activation is likely to be reduced in vivo in subsequent cycles of activity to reduce the negative work production and any associated muscle damage. Despite this, the current model is considered appropriate for measuring HFD‐associated changes in maximal power during repeated muscle activity and is representative of the protocol used in other isolated muscle assessments.

The HFD feeding protocol employed is not without limitation. Rodent models using 45% and 60% HFD have been established as important models for understanding human obesity (Speakman, 2019). Tolerable HFDs in humans can contain up to 60% energy from fat, although western diets typically contain ∼40% (Speakman, 2019). Sixty percent HFD can elicit a more exaggerated metabolic response in rodent models, but differences are often small (Speakman, 2019). Furthermore, data from the present study and from our previous work employing a 45% HFD (Shelley et al., 2024) demonstrate similar impacts on contractile function. The HFD feeding approach employed in the present study, therefore, reflects an appropriate model for understanding the impact of dietary‐induced obesity on skeletal muscle contractile function. However, future work is needed to understand the interaction between dietary macronutrient composition, duration of consumption and age on isolated skeletal muscle function.

The approach employed in the present study should be considered a model for understanding effects of HFD on eccentric muscle function and may not fully reflect the in vivo biomechanical action of the muscle. For instance, during running in mice the SOL functions primarily concentrically and isometrically, although both muscles seem likely to undergo some eccentric activity (James et al., 1995). However, evidence regarding the action of both the EDL and the SOL across a breadth of activities has not been well established, although in HFD‐treated rodents or humans living with obesity, it would seem intuitive to speculate that for each muscle there is an increasing eccentric demand as the requirement for joint stabilisation and deceleration increases in movements that require fast actions and more pronounced joint articulation. The standardised approach to evaluating eccentric muscle function used in the present study provides a pragmatic approach allowing direct comparison between HFD‐ and SLD‐fed animals.

The present work has revealed a number of novel findings but indicates a need for future work to fully understand the effect of HFD on eccentric muscle function. The primary focus of such future work should be to quantify mechanisms causing the disparity between HFD effects on concentric and eccentric modalities. This may currently present some challenges given the limited understanding of eccentric muscle physiology (Herzog, 2018; Hessel et al., 2017); however, an initial avenue for exploration may be to quantify HFD effects on titin isoform expression. Future work examining the reversibility of HFD effects directly on skeletal muscle performance has recently been identified as an area of priority (Tallis et al., 2018), but such work should also look to quantify the effects of body fat reduction on eccentric muscle function. Moreover, although not exclusive to the present study, previous work evaluating HFD effects on isolated muscle function is specific to a single sex, and work is needed to examine if the demonstrated results are sex‐specific.

### 4.4 Conclusion

The present work is the first to demonstrate that HFD effects on eccentric muscle function are muscle‐specific and have little relationship to HFD‐associated changes in either isometric or concentric muscle function. Notably, the EDL displayed a reduction in concentric function following HFD treatment, although eccentric function remained largely unaffected. Conversely, the SOL demonstrated a decrease in eccentric power output relative to muscle mass, despite maintaining maximal concentric power. Furthermore, HFD induced faster concentric fatigue in the SOL. These results demonstrate that HFD may compromise the intrinsic power‐generating capacity of both concentric and eccentric muscle actions, resulting in larger muscles that exhibit relatively poorer performance compared to controls. Collectively, these findings underscore the potential detrimental impact of HFD on skeletal muscle function, which could impair physical performance and exacerbate ill health. Specifically, the impaired or unchanged eccentric function, coupled with elevated body mass, may compromise movement control, and diminish the ability to dissipate impact forces, thereby reducing physical function and increasing injury risk. However, HFD may confer some protective benefits against eccentric‐induced damage of fast‐twitch muscle which may be beneficial for devising eccentric exercise training interventions.

## AUTHOR CONTRIBUTIONS

Jason Tallis and Rob S. James conceived and designed the study. Jason Tallis, Emma L J. Eyre, Derek Renshaw and Josh Hurst performed data collection. Jason Tallis and Sharn P. Shelley analysed the data. Jason Tallis and Sharn P. Shelley prepared the figures. Jason Tallis drafted the manuscript. All authors have read and approved the final version of this manuscript and agree to be accountable for all aspects of the work in ensuring that questions related to the accuracy or integrity of any part of the work are appropriately investigated and resolved. All persons designated as authors qualify for authorship, and all those who qualify for authorship are listed.

## CONFLICT OF INTEREST

The authors declare that the research was conducted in the absence of any commercial or financial relationships that could be construed as a potential conflict of interest.

## FUNDING INFORMATION

None.

## Data Availability

Data can be made available upon request.
